# Supplementary material for: Immunopathogenesis of canine chronic ulcerative stomatitis
Source: PLoS One. 2020 Jan 10;15(1):e0227386. doi: 10.1371/journal.pone.0227386 (PMC6953816; doi:10.1371/journal.pone.0227386)
Supplement: S1 Table — (DOCX) [file pone.0227386.s001.docx]

**S1**. Scoring system for determining the canine ulcerative stomatitis disease activity index (CUSDAI); Total highest score = 32

| **Score** | **1** | **2** | **3** |
| --- | --- | --- | --- |
| Weight loss | <1lb | 1-3lb | >3lb |
| Pain score | 0-1 | 2 | 3 |
| Owner subjective score | Fair attitude, eating | Not eating, lethargic | Suffering |
| # of mucosal ulcers | <4 | 4-6 | >6 |
| Size of ulcers (mm) | <4mm | 5-8mm | >8mm |
| White radiating striae | 1 ulcer | Localized to < 3 sites | >3 sites or generalized |
| Ulcer pseudomembrane | Yes | NA | NA |
| Ulcer associated with missing teeth | Yes | NA | NA |
| White striae associated with missing teeth | Yes | NA | NA |
| Ulcer on attached gingiva | Yes | NA | NA |
| Tongue ulcer | Yes | NA | NA |
| Palate ulcer | Yes | NA | NA |
| Lip ulcer | Yes | NA | NA |
| Glossopalatine arch ulcer | Yes | NA | NA |
| Periodontitis Stage | Stage I | Stage II | Stage III, or 4 |
| Oral bacterial infection | Yes | NA | NA |
| Skin ulcers | Yes | NA | NA |
