# Supplementary material for: Immunopathogenesis of canine chronic ulcerative stomatitis
Source: PLoS One. 2020 Jan 10;15(1):e0227386. doi: 10.1371/journal.pone.0227386 (PMC6953816; doi:10.1371/journal.pone.0227386)
Supplement: S2 Table — (DOCX) [file pone.0227386.s002.docx]

**S2. Case designation, patient sex, CUSDAI score, PD score, and Institution**

| **Case Letter** | **Sex** | **CUSDAI** | **PD score** | **Institution** |
| --- | --- | --- | --- | --- |
| **A** | FS | 25 | 2 | JA |
| **B** | FS | 25 | 2 | JA |
| **C** | FS | 23 | 4 | JA |
| **D** | FS | 13 | 1 | JA |
| **E** | FS | 23 | 4 | JA |
| **F** | MN | ND | 4 | JA |
| **G** | MN | 6 | 2 | JA |
| **H** | FS | 20 | 2 | JA |
| **I** | FS | 10 | 2 | JA |
| **J** | MN | 26 | 4 | JA |
| **K** | F | 12 | 4 | JA |
| **L** | FS | ND | 4 | JA |
| **M** | FS | 14 | 2 | BS |
| **N** | MN | 20 | 3 | BS |
| **O** | MN | 5 | 1 | BS |
| **P** | MN | 24 | 2 | BS |
| **Q** | FS | 18 | 3 | BS |
| **R** | FS | 15 | 1 | BS |
| **S** | MN | 13 | 2 | BS |
| **T** | MN | 14 | 2 | BS |
| **U** | MN | 16 | 2 | BS |
| **V** | MN | 16 | 1 | BS |
| **X** | MN | 24 | 3 | BS |
| **Y** | MN | 15 | 4 | JA |
