# Supplementary material for: Immunopathogenesis of canine chronic ulcerative stomatitis
Source: PLoS One. 2020 Jan 10;15(1):e0227386. doi: 10.1371/journal.pone.0227386 (PMC6953816; doi:10.1371/journal.pone.0227386)
Supplement: S3 Table — (DOCX) [file pone.0227386.s003.docx]

**S3.**

**CLINICAL INVESTIGATION CLIENT CONSENT FORM:**

**Immunopathogenesis of Canine Chronic Ulcerative Stomatitis**

Client name_____________________

Patient name____________________

**Aims and hypotheses**

**Aim:** This study prospectively documents B and T cell populations and other select leukocytes using a slate of special stains and antibody-based assays (immunohistochemistry, confocal immunofluorescence, and direct immunofluorescence) in dogs with CCUS and periodontitis. Normal canine oral mucosa and lymph-node tissue were used as controls

**Hypothesis:** We hypothesized that dogs with chronic ulcerative stomatitis would exhibit a spectrum of pathologic changes and phenotype of infiltrating leukocytes that would inform lesion pathogenesis and that these changes would differ from inflammatory phenotypes in periodontitis.

For this study, 24 canine cases of each category will be enrolled.

I understand that Dr. Jamie G. Anderson at Sacramento Veterinary Dental Services is investigating radiographic, clinical chemistry, histopathologic, and microbiologic features of each of these categories of inflammatory diseases.

I understand that my dog has received a clinical diagnosis of canine chronic ulcerative stomatitis or periodontitis, and that several diagnostic procedures will be performed as part of the standard-of-care.

Such procedures will include (but will not necessarily be limited to, depending on the patient’s medical needs): routine preoperative clinical pathology testing, intraoral dental radiographs, photographic documentation, microbiologic collection of a plaque sample, and histopathologic analysis of oral lesions.

I understand that some or all of these procedures will be performed under general anesthesia. I understand that in addition to the afore mentioned procedures, that would strictly correspond to standard-of-care, an approximately 3-5mL sample of blood will be obtained and frozen for later analysis, as well a plaque sample from the lesion will be collected (also frozen for later analysis), finally, an approximately 10 mm in diameter sample of affected oral tissue will be obtained. For chronic ulcerative stomatitis cases the sample will be cut in three and placed in: 1) RNA later, 2) saline, and 3) formalin. For periodontitis cases, extracted teeth will be placed in formalin.

Because blood samples and biopsies of oral tissues are normally collected as part of the standard of care during the diagnosis of chronic canine ulcerative stomatitis, and oral neoplasms; collection of a small amount of blood and tissue for research purposes will not impose additional risk. Utilization of extracted teeth in periodontitis cases comes at no risk.

I understand that my pet will be humanely treated at all times and all investigative procedures will be performed using the customary methods applied to all other client-owned animals at Sacramento Veterinary Dental Services.

I consent to the use of acquired data for educational and research purposes or publication, as well as the taking of photographs for treatment, research and teaching purposes, provided that neither my pet nor I are identified in any publication reports without my written authorization.

I understand that participation in the study is entirely voluntary and that I may withdraw

my pet at any time.

I understand that there will be no cost to me for photographs, dental radiographs, evaluating blood samples, microbiology or histopathologic analysis. Any tests or procedures unrelated to this study will be my financial responsibility.

My questions about this clinical trial have been answered to my satisfaction. If I have additional questions about this study I may write, phone, or email the principal investigator at the following:

Dr. Jamie G. Anderson

Sacramento Veterinary Dental Services

2939 Mather Field Road

Rancho Cordova, CA 95670

(916) 368-7626

(707) 344-2328

[jgadvm@gmail.com](mailto:jgadvm@gmail.com)

My signature below indicates that I hereby grant permission for my pet to participate in this clinical trial.

Signature of Owner_____________________________ Date:

Signature of Witness_____________________________
