# Supplementary material for: Immunopathogenesis of canine chronic ulcerative stomatitis
Source: PLoS One. 2020 Jan 10;15(1):e0227386. doi: 10.1371/journal.pone.0227386 (PMC6953816; doi:10.1371/journal.pone.0227386)
Supplement: Attachment — Submitted filename: Response to Reviewers.docx [file pone.0227386.s005.docx]

Response to Reviewer’s comments:

Reviewer #1: The authors sought to investigate some leukocyte populations by examining lesions from dogs with chronic ulcerative stomatitis. Nonetheless, there are many concerns described below:
1. The introduction is too long, and just in this section the authors cited 45 articles. I strongly suggest the authors to be concice.

The introduction is now significantly reduced in length; we anticipate that the Reviewer will find it more concise. We have further reduced the number of published articles cited throughout this section (11).

2. Line 241. Why the authors used HE or toluidine blue? Why the authors did not standardize the dye used to stain mucosa tissue?

We thank the Reviewer for highlighting this source of confusion. We stained all tissue samples with H&E (hematoxylin and eosin) and toluidine blue. HE staining was used for routine histopathology, while toluidine blue was used to highlight mast cell granules which can be difficult to identify with H&E. Toluidine blue-stained tissue sections were utilized to quantify the number of mast cells within the tissues. We have corrected the text accordingly (see lines 91-149 in the revised manuscript)

3. One of my major concerns are the antibodies used in immunohistochemistry.

We thank the Reviewer for the opportunity to clarify these critical issues. As veterinary clinician-scientists, the authors are very familiar with the challenges that are associated with the paucity of well validated antibodies generated against the antigens of non-standard animal species. We have provided detailed information about each antibody that was used in this work. All of the antibody details have been summarized in a table format (see Table 1 in the revised manuscript) highlighting the specific antibodies that we have used throughout the study, including their catalogue number and citations to validate their use in dogs.

What is the specificity of the antibodies used for dogs?

All antibodies used in this study are either made against canine antigens (i.e. CD4, CD8), or were previously validated to cross react with canine antigens (CD3, CD20, CD204, FoxP3, Mum-1 and IL17) . Please see Table 1 for details.

The cross-reactivity of all antibodies used must be proven (e.g. anti-CD20, anti-Mum1 and anti-CD204)

We have included citations that previously determined the cross reactivity and validity of the use of these antibodies against the canine antigens (see Table 1 for details).

How the authors titrate the antibodies? All antibodies used to identify leukocyte populations are monoclonal antibodies (e.g. anti-CD20)? This information should be clearly stated.

Antibodies were titrated either in our research labs (FoxP3, IL17), or by the histopathology laboratory of the UC Davis, Veterinary Medical teaching Hospital (CD3, CD20, Mum-1 and CD204). Antibodies were titrated to a point where specific target cell staining was most evident. In contrast to flow cytometry, where the positive and negative signals may be quantified, and difference may be objectively optimized, in microscopy based method, objective signal quantification is not typically performed.

Why the authors did not include a negative control in immunohistochemistry assay? The controls of immunohistochemistry should be carefully conducted (please, for more details see: Hewitt et al., J Histochem Cytochem. 2014).

All immunohistochemistry and immunofluorescence studies had several layers of negative and positive controls to ensure appropriate interpretation of our findings. Specifically, we have used a ‘No antibody’ control to determine the level of tissue auto-fluorescence and ‘No primary antibody control’ to determine the level of non-specific secondary antibody binding. For positive controls we have used canine lymph node sections which contain the various immune cell subsets. Moreover, cell subset distribution within the lymph node tissue was further used to evaluate our positive controls (see line 276-282 in the revised manuscript).

How the authors select their antibodies?

Why the authors used CD20 to identify B cells ("CD20 is expressed primarily on B cells but has also been detected on both normal and neoplastic T cells" available at <https://www.thermofisher.com/order/catalog/product/RB-9013-P0#/RB-9013-P0?> Why the authors used anti-CD204 (a macrophage scavenger receptor) antibody to identify macrophages?

As the Reviewer indicates, when choosing an antibody for use in a non-standard animal species based research project, one has to consider not only the antigen biology and its advantages and disadvantages as a specific cell marker, but also the cross reactivity and validity of the use in the specific animal species, in our case, the dog. All of the antibodies that we have used in this study were either generated specifically against the canine antigen (i.e. CD4 and CD8), or were previously validated to be cross reactive. CD3 is a protein complex that is uniquely expressed on the cell surface of almost all T cells and is responsible for delivering the signal upon T cell receptor ligation by its cognate antigen. CD3 is widely used as a pan T-cell marker. FoxP3 is a master regulator transcription factor that is expressed in T regulatory cells, though it may be induced in other T cell subsets as well [CIT]. CD4 is a widely accepted marker of T helper cells and CD8 is a widely accepted marker for cytotoxic T cells. CD20 is membrane bound protein with a yet to be determined function, that is expressed throughout the developmental stages of B cells, excluding the early pro-B cells and plasma cells. Rarely, CD20 may be faintly expressed on T cells. To ensure that we correctly capture plasma cells that do not express CD20, we also stained for Mum-1 (aka interferon regulatory factor 4), a transcription factor that is specifically expressed in plasma cells(1, 2). Our CD3 staining was further used to ensure that our CD20 staining, does not label T cells, as well as B cells. CD204 is a scavenger receptor (aka Scavenger receptor A) that is expressed on the cell surface of macrophages(3, 4).

4. Please, provide clearly the information about the monoclonal antibodies used to identify CD4+ and CD8+ cells. It is also a pity that CD4 and CD8 antibodies were not used together. Unfortunately, the importance of T CD4 CD8 double negative (e.g Fisher et al., Blood, 2005; D'Acquisto, Biochemical Phamarcology, 2011) and even positive (e.g. Clénet et al., Sci. Rep., 2019) cells was neglected in the present study. Furthermore, it would be great if the macrophage subsets has been considered.

Please see the previously mentioned Table 1 for specific details regarding the antibodies directed against CD4 and CD8. While co-labeling of tissues with CD4 and CD8 might have allowed us to look at rare double negative or double positive T cell populations, both antibodies are made in mouse, thus multiplexing is not feasible. Based on our subjective evaluation, it seems like the great majority of the CD3+ T cells were either CD4 or CD8 positive, though direct evaluation could not be performed.

5. The cross-reactivity of anti-human IL17 antibody must be determined/shown. The specifications of all antibodies used should be clearly stated (if possible, include the catalog number).

The goat anti-human IL17 antibody that we have used has been previously validated for use in dogs by one of the co-authors of this manuscript [Kol et al. Th17 pathway… PLOs One 2016]. Please see above for citation. We have compiled and provided a table that includes all information regarding the antibodies that were used throughout this study.

6. It is a pity that the authors did not quantify IL-17 by qPCR in tissue, and the producing cells is not clearly identified.

We agree that transcriptional based research will provide an additional layer of information to elucidate the pathophysiology of this frustrating canine disease. As mentioned in the Materials and Methods of the manuscript, lesional tissues were subdivided and a sample stored in RNAlater was archived for future transcriptional studies. We are now pursuing a non-biased transcriptional characterization of tissues from affected dogs via RNA-seq approach, followed by qRT-PCR and RNA-scope to localize transcripts within the tissue and associate them with specific cell lineage. We plan to report our findings in a future manuscript.

7. Line 389-390. The PloS One rules recommended that al data should be clearly showed.

All data has now been provided as required by the journal guidelines in Supplement D.

8. The conclusion section should be profoundly revised.

The authors think that the short ‘Conclusion’ paragraph summarizes our main findings in this study. We would be happy to consider specific comments and suggestions raised by the Reviewer.

Reviewer #2: This was a well designed and rigorously performed analysis of the pathological processes in this frustrating disease of the canine oral cavity. It is good to see mounting evidence of the likely immune-mediated pathway, in order that further research can hopefully lead to better methods of control or cure.

Cited literature:

1.	Ramos-Vara JA, Miller MA, Valli VE. Immunohistochemical detection of multiple myeloma 1/interferon regulatory factor 4 (MUM1/IRF-4) in canine plasmacytoma: comparison with CD79a and CD20. Vet Pathol. 2007;44(6):875-84.

2.	Stilwell JM, Rissi DR. Immunohistochemical Labeling of Multiple Myeloma Oncogene I/Interferon Regulatory Factor 4 (MUMI/IRF-4) in Canine Cutaneous Histiocytoma. Vet Pathol. 2018;55(4):517-20.

3.	Kato Y, Murakami M, Hoshino Y, Mori T, Maruo K, Hirata A, et al. The class A macrophage scavenger receptor CD204 is a useful immunohistochemical marker of canine histiocytic sarcoma. J Comp Pathol. 2013;148(2-3):188-96.

4.	Wagner A, Junginger J, Lemensieck F, Hewicker-Trautwein M. Immunohistochemical characterization of gastrointestinal macrophages/phagocytes in dogs with inflammatory bowel disease (IBD) and non-IBD dogs. Vet Immunol Immunopathol. 2018;197:49-57.
